# Study of double button plate and cannulated screw fixation for posterior cruciate ligament avulsion fracture

**DOI:** 10.3389/fsurg.2022.887010

**Published:** 2023-01-13

**Authors:** Kai Sun, Meng Fan

**Affiliations:** Orthopaedic of Tianjin First Central Hospital, Tianjin, China

**Keywords:** posterior cruciate ligament, avulsion fracture, double button plate fixation, cannulated screw fixation, treatment

## Abstract

**Background:**

The posterior cruciate ligament (PCL) plays an important role in maintaining the stability of the knee joint. To date, researchers have not reached agreement on which type of ﬁxation material should be used to treat PCL tibial avulsion fractures. The aim of this study was to investigate the effects of double button plate and cannulated screw fixation in the treatment of PCL avulsion fractures.

**Methods:**

We retrospectively reviewed our database, which was collected prospectively. From January 2019 to January 2020, 46 patients with posterior cruciate ligament avulsion fractures who were treated with double button plate and cannulated screw fixation. The primary outcomes of this study were surgical complications (fixation failure/displacement, implant breakage, nonunion, infection), radiological parameters, and knee function and secondary outcomes included reoperation rates for the fixation methods and the prevalence of symptomatic hardware causing soft tissue irritation outcomes were included. Values were analysed using multiple comparisons, where *P*-values of 0.05 or less were considered significant.

**Results:**

Double button plate fixation had significantly higher values than cannulated screw fixation. The results showed that double button plate fixation was related to greater decreases in the length of surgery, intraoperative blood loss, hospital days, full weight bearing time, and incidence of complications, as well as greater increases in postoperative range of motion and Knee Society Score function and Lysholm scores.

**Conclusion:**

Compared with cannulated screw fixation, the use of double button plate fixation technology has the following advantages: less trauma, shorter operation time, convenient use of instruments and fixtures, and it does not need to be removed, thus avoiding secondary trauma. Moreover, double button plate fixation under direct vision is safe and reliable without the need for additional equipment.

## Introduction

The posterior cruciate ligament (PCL) plays an important role in maintaining the stability of the knee joint (1). In general, simple PCL injury is not common, and tibial avulsion fracture is the most common type. It has been reported that avulsion fracture of the tibial insertion is common in young people. The main causes of avulsion fractures are traffic accidents and falling injuries during strenuous exercise (2). The damage mechanisms usually include overflexion, anteroposterior and hyperextension. PCL tibial avulsion fracture is a special type of PCL injury, accounting for approximately 10% of all PCL injuries (2, 3). In the past, due to insufficient understanding and limited examination instruments, misdiagnosis and missed diagnosis led to untimely treatment, poor treatment effect, a high risk of fracture displacement, delayed union and nonunion, and dysfunction of the knee joint in later stages (4).

The treatment of PCL tibial avulsion fractures includes nonoperative treatment, traditional open reduction internal fixation, and arthroscopic surgery (4). There is no consensus on the treatment of PCL tibial avulsion fractures in the past.Some scholars (5) believe that the strength of quadriceps femoris can be enhanced through rehabilitation exercise to compensate part of PCL function. Some studies (6) advocate that the PCL tibial avulsion fractures can be fixed with plaster if the displacement does not exceed 10 mm and the rotation does not exceed 5°. Most people believe that conservative treatment can be chosen for those with displacement less than 3 mm, and surgical treatment is recommended for those with displacement greater than 3 mm or turnover of fracture fragment. However, with the increase of the number of cases and the continuous progress of research, it is recognized that there will be compression and incarceration in the fracture fragment, which will affect the fracture reduction and healing. Therefore, nonoperative treatment will lead to delayed healing and even bone nonunion, which will eventually lead to PCL dysfunction, and affect the stability of knee joint.

Open surgery has been widely performed, and the main internal fixation materials include absorbable screws, cannulated screws, and anchors with threads. Cannulated screw fixation has high fixation strength and stability, but all the stress is concentrated in the tail of the screw, which easily causes re-fracture and leads to internal fixation failure; at the same time, the huge tension of the PCL during knee joint movement may lead to higher risks of re-fracture and fracture block displacement, limiting early functional exercise and increasing complications. Arthroscopic internal fixation can complete the exploration of the knee joint through several small incisions and monitor the operation in the process of drilling the tunnel or screw placement. While this method has the advantages of minimal surgical trauma and fast patient recovery, it also features high technical requirements for surgeons, a long learning curve, high equipment requirements, and expensive equipment (7). At present, open surgery and internal fixation remain mainstream. The appearance of internal fixation materials has given surgeons more choices; for example, the fracture block can be fixed directly through a posterior small incision.

However, internal fixation methods have many disadvantages, such as insufficient fixation strength, high tension on the cruciate ligament and long-term immobilization, which to some extent affect early postoperative rehabilitation exercise, resulting in knee joint adhesion, flexion and extension function limitations and other shortcomings (7). An alternative method is to utilize double button plate fixation. We adopted double button plate fixation in the management of PCL avulsion fractures. To date, researchers have not reached an agreement on which type of ﬁxation material should be used to treat PCL tibial avulsion fractures. Therefore, this study aimed to evaluate the associations of perioperative and postoperative outcomes between double button plate and cannulated screw fixation.

## Materials and methods

### Patient demographics and assessment

**Figure 1:**
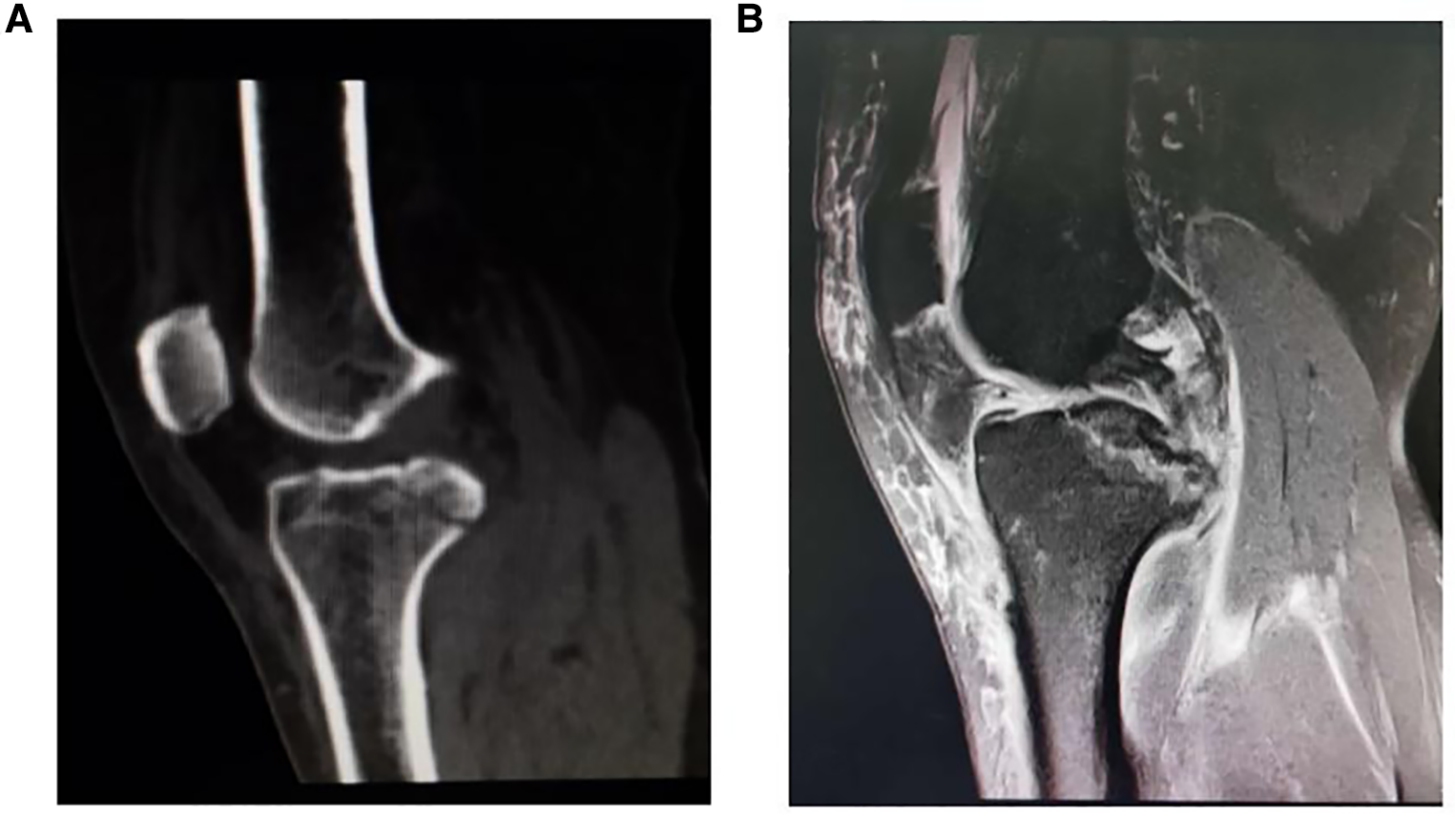
Preoperative radiograph of posterior cruciate ligament (PCL) avulsion fracture. (**A**) CT scanning showed the posterior cruciate ligament avulsion fracture. (**B**) MRI confirmed the fragment size and displacement of the PCL avulsion fracture.

**Figure 2:**
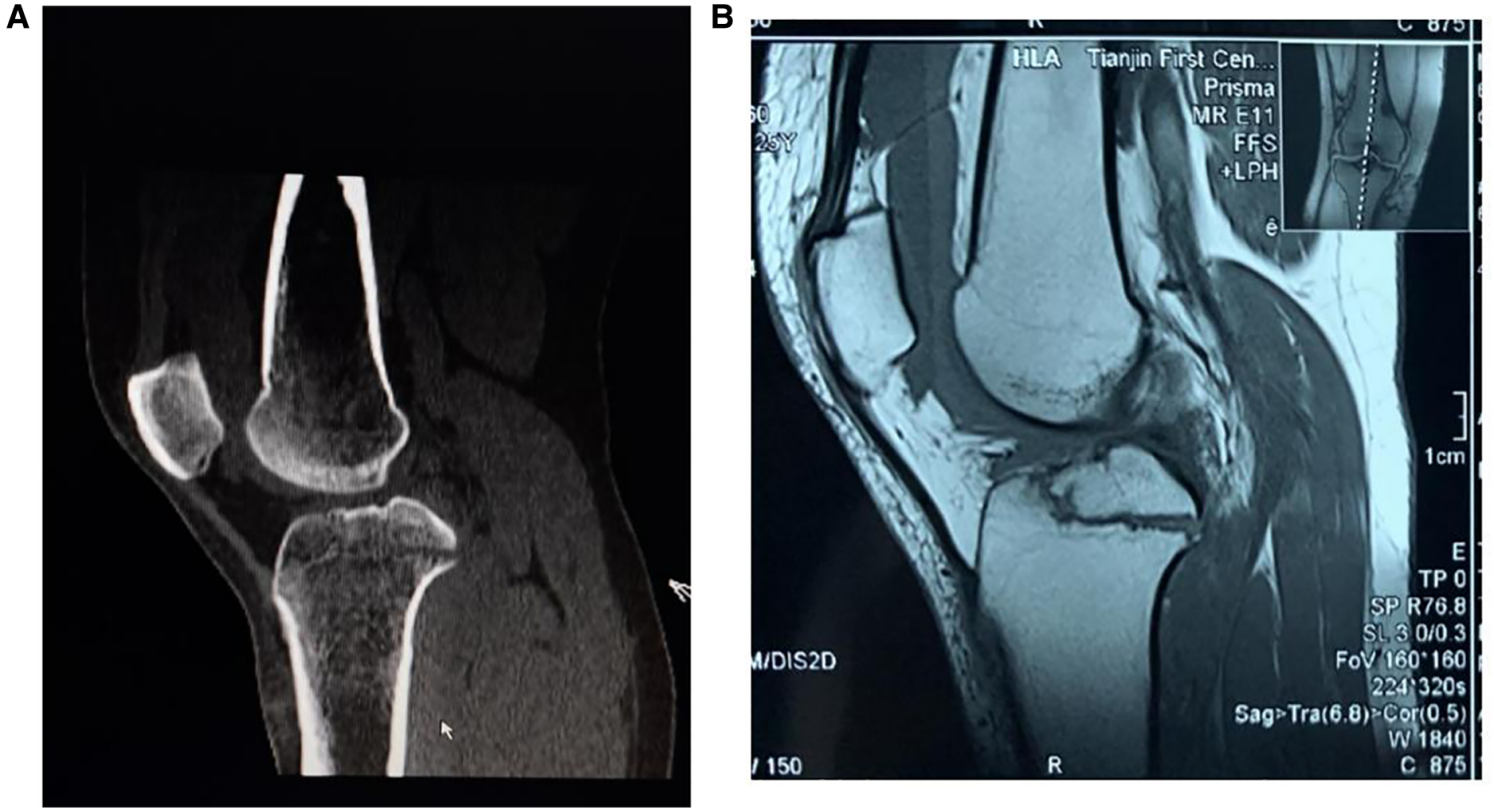
Preoperative radiograph of posterior cruciate ligament (PCL) avulsion fracture. (**A**) CT scanning showed the posterior cruciate ligament avulsion fracture. (**B**) MRI confirmed the fragment size and displacement of the PCL avulsion fracture.

We retrospectively reviewed our database, which was collected prospectively. From January 2019 to January 2020, 46 patients had PCL avulsion fractures. The patients signed an informed consent form approved by the Institutional Review Board at our hospital (Tianjin First Central Hospital; No. 0891). The clinical data of 46 patients with regular follow-up were obtained, including 23 cases of double button plate fixation, with 12 males and 11 females, and 23 cases of cannulated screw fixation, with 11 males and 12 females. The inclusion criteria were a single avulsion fracture of the PCL, time of injury less than 3 weeks, complete imaging examination of the knee joint, positive posterior drawer test and Lachman test, and type II or type III according to the Meyers classification. The exclusion criteria were combined tibial plateau fracture, fracture around the knee joint, comminuted fracture, pathological fracture, Meyers type I, anterior and PCL injuries, medial and lateral collateral ligament injuries, and combined severe vascular and nerve injuries. Two groups of patients were randomly assigned. Traffic accidents were the major mechanism of injury, accounting for 34 (74%) of 22 cases, along with 8 cases of fall injury (17%) and 4 cases of injury involving accidental sprains (9%) (Table 1, Figures 1, 2).

**Table 1:** Patient demographics.

Demographics	Double button plate fixation	Cannulated screw fixation	*P*
No. patients	23		23		N/A
Sex (male:female)	12:11		11:12		N/A
Mean age (years)	38.5 ± 15.5		37.5 ± 16.5		>0.05
Injury	Traffic accidents 18 Fall injuries 3 Accidental sprains 2		Traffic accidents 16 Fall injuries 5 Accidental sprains 2		N/A
Average OT (minutes)	45 ± 10.5		55 ± 20.5		<0.05
Average BL (ml)	25 ± 10.5		30 ± 25.5		>0.05
Average HST (days)	5		7		N/A
Comparison of scores					*P*
ROM (degree)	Preoperative Postoperative		Preoperative Postoperative		<0.05
	0–50	0–125	0–55	0–120	
KSS function (scores)	Preoperative Postoperative		Preoperative Postoperative		<0.05
	40	90	40	70	
Lysholm (scores)	Preoperative Postoperative		Preoperative Postoperative		<0.05
	40.5 ± 8.5	92.5 ± 5.5	35.5 ± 6.0	90.5 ± 3.5	
FWB (weeks)	11		14		N/A
Nonunion	NO		NO		N/A
Any infection	NO		NO		N/A
Revision	NO		1		N/A
Implant removal	NO		4		N/A

M, male; F, female; OT, operation time; BL, blood loss; HST, hospital stay time; ROM, Range of motion; KSS, Knee Society Score; FWB, full weight bearing; N/A, not applicable.

### Surgical technique

**Figure 3:**
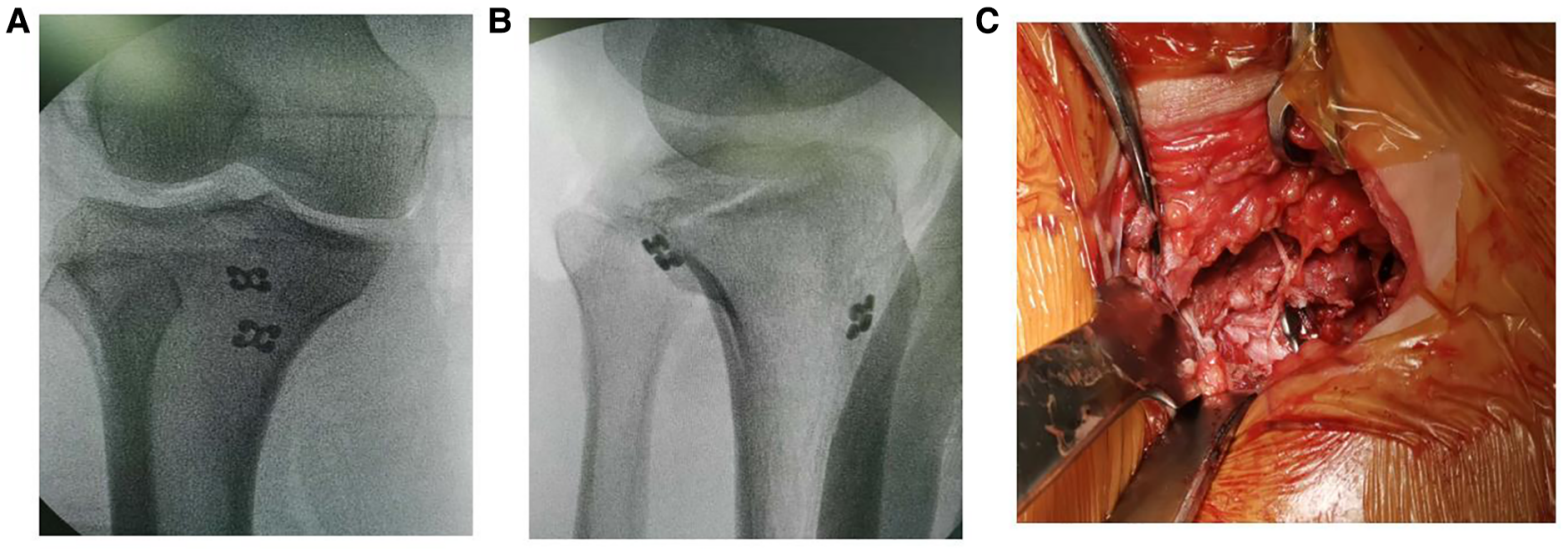
Intraoperative radiograph of double button plate fixation (**A**) and (**B**) intraoperative x-ray showed that the fracture was well reduced and fixed, (**C**) showed the titanium plate passed bone canal and position was acceptable.

We performed all procedures using general anesthesia with the patient in the prone position. We examined knee range of motion and laxity with the patient under general anesthesia, and the injured leg was bound with a tourniquet. A 6-cm-long longitudinal incision was made. We protect the popliteal blood vessels and nerves, separate the muscle space, clear the joint cavity, explore the PCL tibial avulsion fracture (Figure 3C).

### Double button plate fixation group

After reduction, Kirschner wire was used for temporary fixation. We established the bone canal of the posterior cruciate ligament with a guide needle of a diameter of 2 and 4.5 mm hollow drill, passed double button plate(Suture Anchor, Arthrex). from the bone canal, and tighten a knot behind the fracture. We weaved the ligament side with non absorbable suture (Figure 3C) and keep it from unfurling. Physical examination showed that the knee joint was stable. Intraoperative radiograph showed that the fracture was well reduced and fixed, and the position of the titanium plate was acceptable (Figures 3A,B). The leg was fixed with plaster at 10 degrees of extension.

### Cannulated screw fixation group

**Figure 4:**
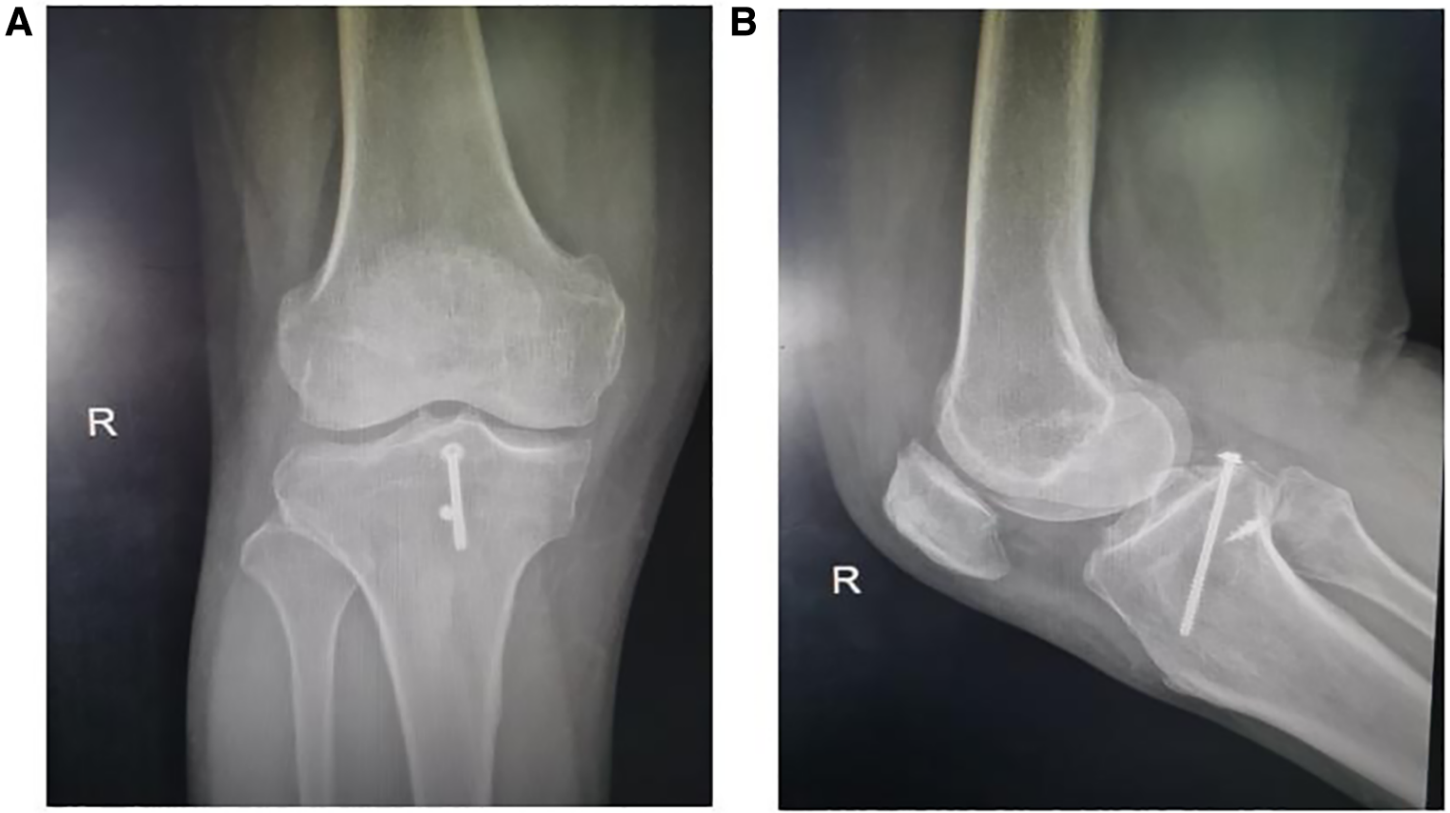
Postoperative radiograph of cannulated screw fixation (20 months). Follow-up x-rays showed that the avulsion fracture fragments of the tibial attachment point of the PCL healed well.

The avulsion fracture block was exposed, the blood clot at the fracture end was cleaned, and the knee was bent at 30° to maintain the anterior drawer position of the proximal tibia. The guide pins were crossed vertically with the fracture surface towards the tibial tuberosity direction for temporary fixation; along with guide pin sounding and tapping, a cannulated screw with a diameter of 3.5 mm was tightened with a gasket. A metallic suture anchor fixed the ligament avulsion fracture. C-arm fluoroscopy showed that the avulsion fracture fragment of the PCL attachment point was well reduced, and the cannulated screw position and length were suitable (Figures 2, 4). The guide needle was removed, and the limb was fixed with plaster at 10 degrees of extension.

### Postoperative protocol

**Figure 5:**
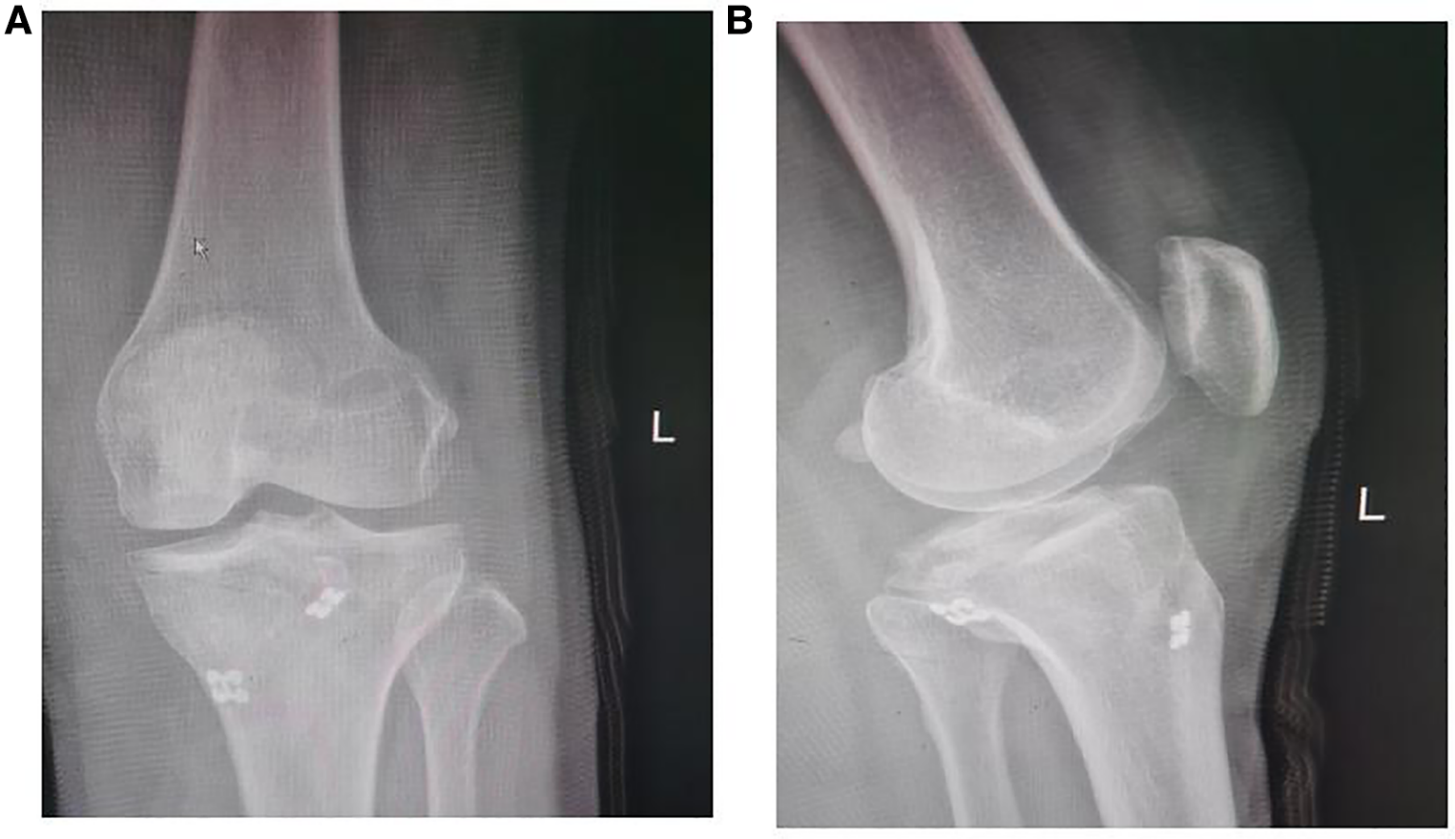
Postoperative radiograph of double button plate fixation (20 months) cannulated screw fixation. Follow-up x-rays showed that the avulsion fracture fragments of the tibial attachment point of the PCL healed well, and there was displacement or implant breakage of the fracture fragments.

Postoperative radiographs were taken after surgery (Figures 4, 5), and patients were given a plaster as protection. In general, 0–90 degrees of flexion in the brace was allowed for the first 2 weeks postoperatively. Patients attended their first follow-up appointment at 2 weeks postoperatively for wound inspection, and then they were followed up every 2–4 weeks to be monitored for functional return and clinical/radiological fracture union (Table 1). The knee joint activity gradually strengthened by the fourth week after the operation, the passive flexion activity was 120°, and some weight-bearing walking began at the sixth week after surgery. Weight-bearing walking was completely carried out 8 weeks after the operation, and the injured state was essentially restored at 12 weeks after the operation.

### Statistical analysis

Data were analysed using SPSS 13.0 software (SPSS, Chicago, IL, USA). All data are reported as the mean ± standard deviation (SD). One-way ANOVA was employed for all statistical analyses, followed by the Student-Newman-Keuls test. Values were analysed using multiple comparisons, where *P*-values of 0.05 or less were considered significant.

## Results

At 2 weeks after the operation, the plaster was removed. All patients had normal flexion and extension of the knee joint at 6–8 weeks; and 3 months after the operation, the fractures had reached the healing standard. Functional outcomes were assessed by means of walking status, range of motion, and KSS function and Lysholm scores upon final follow-up (Table 1).

The average preoperative scores of two groups were both 40 points by KSS function scores, double button plate 40.5 vs. cannulated screw fixation 35.5 points by Lysholm scores. Average clinical outcome scores improved significantly at the final follow-up (mean, 20 months; range, 16–24 months) after surgery, double button plate 90 points vs. cannulated screw fixation 70 points by KSS function scores, double button plate 92.5 points vs. cannulated screw fixation 90.5 points by Lysholm scores (*P*  <  0.05) (Table 1). All patients returned to work at the final follow-up. Knee active range of motion improved significantly, double button plate 75° vs. cannulated screw fixation 65° for external rotation. There were no cases of infection, neural injury, suture anchor problems.

The primary outcomes of this study were surgical complications (fixation failure/displacement, implant breakage, nonunion, infection), radiological parameters, and knee function. Secondary outcomes included reoperation rates for the fixation methods and the prevalence of symptomatic hardware causing soft tissue irritation.

Overall, the average time to final follow-up was 20 months (range, 16–24 months), and the fractures had healed 3 months after the operation. The fractures were not displaced except in one case in the cannulated screw fixation group, and there were no neurovascular fractures or infections. Follow-up x-rays showed that the avulsion fracture fragments of the tibial attachment point of the PCL healed well, and there was no displacement or implant breakage of the fracture fragments. One patient in the cannulated screw fixation group underwent reoperation after initial fracture fixation (Table 1). Four patients in the cannulated screw fixation group requested implant removal due to symptomatic discomfort in the knee.

## Discussion

The tension of the PCL is highest in the flexion position of the knee joint, and tibial avulsion fractures mainly occur in knee flexion. In the flexion position, the lateral condyle of the femur moves backwards, and the femur rotates outwards. The force from front to back causes the posterior tibia to be impacted by the femoral condyle, and the tension of the PCL increases sharply, which eventually leads to avulsion fracture of the PCL tibial insertion point (8). Because the contact area between the posteromedial tibia and the medial femoral condyle is larger than that between the posterolateral tibia and the lateral femoral condyle, PCL tibial avulsion fracture fragments are increasingly larger. If not intervened, they can seriously affect the stability of the posterior part of the knee joint, and the fracture block protrudes behind the tibial intercondylar ridge, which may induce impingement syndrome (9). In the late stage, quadriceps atrophy, joint effusion, articular cartilage degeneration and secondary meniscus injury can become aggravated, which will seriously affect the quality of life of patients. Therefore, the current view is that PCL tibial avulsion fractures should be firmly fixed to avoid postoperative displacement or poor healing, resulting in instability of the knee joint (10).

There are many surgical methods for PCL tibial avulsion fractures, including arthroscopic and open surgical approaches. Arthroscopic surgery has the advantages of minimal trauma, a small amount of soft tissue damage and fast recovery. However, in most total arthroscopic surgeries, a bone tunnel must be drilled on the tibia. An internal fixator is introduced through the tunnel, which has the characteristics of a complex operation, high equipment requirements and a long learning time, thus limiting its development (11). Open surgery for PCL tibial avulsion fractures has been widely carried out. The internal fixation materials mainly include steel wires, absorbable screws, hollow screws, Kirschner wires, and wire anchors. Steel wire fixation is convenient and economical, but it easily causes further fracture of the fracture block in the process of the operation, causing secondary damage to the blood supply of the fracture block and affecting fracture healing; moreover, to avoid the fracture of the steel wire after the operation, long-term braking is necessary, resulting in limited joint function and joint stiffness (12). The absorbable screw material can be absorbed after the operation, and it is unnecessary to remove the internal fixator, avoiding a secondary operation and trauma. However, the external fixation time of absorbable screws, such as plasters or braces, is long, and approximately 4–6 weeks of fixation is advocated. At the same time, absorbable screws have low strength and are characterized by weak fixation and easy displacement (9). Simple cannulated screw fixation has high fixation strength and stability, but all the stress is concentrated in the screw tail, which easily leads to stress concentration, refracture and internal fixation failure (12).

Due to comminuted fracture, small bone fragments are often difficult to fix, but early rehabilitation exercise requires relatively stable fixation. For the treatment of this kind of fracture, in addition to conventional surgical methods such as tension band steel wire fixation, purse suture fixation and special plate fixation are also currently commonly used. For patients with PCL avulsion fracture and repair difficulties, a more classic treatment method is to insert two button plates into the broken end of the fracture. The sutures are sewn out along the aponeurosis, and then the two circuits are sewn and knotted with each other. With a button plate, the fracture block is fixed reliably, the fracture surface is in good contact, and the PCL is connected by a nonabsorbable tension suture. This method can reduce any large movement of the fracture block during knee joint functional exercise, disperse the concentrated stress around the button plate during knee joint flexion, reduce the risk of refracture, promote fracture healing and avoid joint adhesion complications (11–16).

Compared with cannulated screw fixation, the use of double button plate fixation technology has the following advantages: less trauma, shorter operation time, convenient use of instruments and fixtures, and it does not need to be removed, thus avoiding secondary trauma. Moreover, the strength of the suture is great, and the tension of the PCL can be fully reduced by tying the suture band. Double button plate fixation under direct vision is safe and reliable without the need for additional equipment. Even comminuted fractures can be effectively fixed by knotting.

### Limitations

There are some shortcomings in this study, such as the small sample size, which may lead to deviations in the evaluation of curative effects. Further expansion of the sample size and long-term follow-up are needed to confirm this.

## Conclusion

In this study, double button plate fixation technology for the treatment of PCL avulsion fractures has the advantages of minimal trauma, a simple operation, reliable fixation and a large fracture contact area, fewer postoperative complications, and restored stability and function of the knee joint. Our results suggest that this reconstruction technique is a reliable and useful alternative treatment.

## Data Availability

The raw data supporting the conclusions of this article will be made available by the authors, without undue reservation.
